# Radical Cystectomy with Elective Indication to Cutaneous Ureterostomy: Single-Center Comparative Analysis Between Open and Robotic Surgery in Frail Patients

**DOI:** 10.1590/S1677-5538.IBJU.2024.0556

**Published:** 2025-01-22

**Authors:** Maria Chiara Sighinolfi, Enrico Panio, Tommaso Calcagnile, Simone Assumma, Filippo Gavi, Luca Sarchi, Matti Sagalli, Filippo Turri, Alberto Romano, Alberto del Nero, Paolo dell’Orto, Marco Sandri, Andrea Gregori, Franco Palmisano, Bernardo Rocco

**Affiliations:** 1 Fondazione Policlinico Universitario Agostino Gemelli IRCCS Department of Urology Rome Italy Department of Urology, Fondazione Policlinico Universitario Agostino Gemelli IRCCS, Rome, Italy; 2 ASST Santi Paolo and Carlo Department of Urology Milan Italy Department of Urology, ASST Santi Paolo and Carlo, Milan, Italy; 3 University of Brescia Big Data and Statistics Brescia Italy Big Data and Statistics, University of Brescia, Brescia, Italy; 4 San Gerardo Hospital Department of Urology Monza Italy Department of Urology, San Gerardo Hospital, Monza, Italy

**Keywords:** Ureterostomy, Cystectomy, Robotic Surgical Procedures

## Abstract

**Objectives:**

Radical cystectomy (RC) is a surgical procedure associated with high rates of morbidity. The aim of the study is to provide a comparison between robotic (RARC) and open RC (ORC) in patients elected to cutaneous ureterostomy (CUS).

**Materials and Methods:**

This is a retrospective single-center cohort study performed at a high-volume institution. The study involved 64 patients undergoing RC with CUS, 42 ORC and 22 RARC. The indication for RC was based on EAU guidelines and the choice of CUS was planned due to advanced oncological stage or patient's frailty. Patient allocation to the robotic or open approach for RC was casual, determined by surgeon preference and/or the availability of a robotic operating room. The Adverse events were systematically graded utilizing the Clavien–Dindo classification system.

**Results:**

Complications of Clavien Dindo ≥ 2 occurred in 27 out of 42 (64.2%) ORC and 3/22 (13.6%) RARC (p < 0.001); complications of Clavien Dindo ≥ 3 occurred in 10/42 (23.8%) ORC and only 1/22 (4.5%) RARC, respectively (p = 0.08). Multivariable analysis revealed that robotic surgery was the only variable inversely associated with Clavien Dindo ≤ 2 complications.

**Conclusions:**

In conclusion, RARC appears to be associated with lower morbidity and reduced incidence of complications, elements that make it particularly suitable for frail patients with an elective indication for CUS.

## INTRODUCTION

Radical cystectomy (RC) is a surgical procedure associated with high rates of morbidity and non-negligeable mortality (1). The use of the robotic approach was firstly introduced by Menon et al. in 2003 (2). Thereafter, robotic-assisted radical cystectomy (RARC) has surged worldwide after several randomized controlled trials (RCTs), that stated its non-inferiority to the open approach in terms of oncological results (3–6). For example, the multi-institutional RAZOR trial confirmed similar 2-year progression-free survival rate, 3-year recurrence-free, progression-free survival, and overall survival between RARC and open RC (ORC) (5). As far as post-operative results are concerned, a reduction in morbidity is expected with the robotic approach. Systematic reviews, retrospective, prospective and RCTs reported shorter lengths of hospital stays (LOS) and reduced transfusion need after RARC, if compared to ORC (7–12). Ileal conduit and neobladder have been both applied in robotic surgery, either within an extracorporeal and intracorporeal approach (13). Nevertheless, for some patients, cutaneous ureterostomy (CUS) may be preferred because they result to be faster and simpler than other diversion types: the lack of bowel use may reduce operative time, intensive care unit (ICU) requirement, and incidence of perioperative complications, especially those related to intestinal manipulation (14). CUS are often applied in case of locally advanced disease: in those patients, a salvage cystectomy could be performed to debulk the disease and relieve symptoms such as bleeding (14, 15). While previous evidence focused on the comparison between robotic and open surgery with the reconfiguration of a urinary intestinal diversion, to now the evaluation of robotic and open RC with CUS has never been addressed. The aim of the paper is to provide a single center comparison between RARC and ORC in patients selectively elected to CUS.

## MATERIALS AND METHODS

This is a retrospective single-center study on a prospectively maintained dataset of patients treated with ORC or RARC and CUS from January 2022 to September 2023 at ASST Santi Paolo and Carlo, Milan, Italy. This study was conducted and reported in accordance with the STROBE guidelines for observational research (16). Patient allocation to the robotic or open approach for radical cystectomy was casual, determined by surgeon preference and/or the availability of a robotic operating room. The study involved patients elected to RC with upfront selection of CUS as urinary diversion.

The choice of CUS was pre-operatively planned and deemed necessary due to the following condition/s:

- Advanced clinical oncological stage defined from pre-operative CT scan or MRI.
- Patient's frailty as graded by the ASA score and/or Charlson Comorbidity Index (CCI) and/or anesthesiologic indication to short operative time.

Inclusion criteria were age ≥ 18 years and a diagnostic TURBt for BCa with a T stage (cT) 2-3 or recurrent BCG failure high-grade cancer with indication to RC according to the EAU Guidelines (17). All patients were informed about the diversion type and signed informed consent to the procedure. Exclusion criteria were the simultaneous resection of other organs (including nephroureterectomy) and missing follow up data. RARCs were carried out with either Da Vinci or Hugo RAS system. All surgeries were performed by experienced operators (B.R., F.T., P.D.O. and A.R.) with extensive experience in the approach they chose.

### Pre-operative evaluation

Intestinal preparation was preoperatively omitted. The implementation of an Enhanced Recovery After Surgery (ERAS) protocol was undertaken, whether deemed feasible (18). Notwithstanding, prompt mobilization within the 24-hour postoperative window and initiation of enteral nutrition were encouraged. Concurrently, prophylactic administration of low molecular weight heparin was kept for a duration of four weeks in accordance with established clinical guidelines. Postoperative management included the removal of the abdominal drain on 1-2 PO days; ureteral catheters were left and changed every 2-3 months thereafter. Adverse events were systematically graded utilizing the Clavien-Dindo classification system (18). Routine post-discharge follow-ups were conducted on a weekly basis, extending up to 30 days post-discharge.

### Surgical technique for RARCs

The patient is positioned in a standardized 21° Trendelenburg inclination. The docking is performed according to the robotic system as previously described (19, 20). Then, bilateral identification and isolation of the ureters are executed, and extended superiorly from the iliac vessels until their respective insertions into the bladder. Subsequent ligation and transection of the ureters are performed using a median-sized Hem-o-lok clip. In male subjects, the dissection is initiated at the level of the seminal vesicles (SV), creating a surgical plane between Denonvillier's fascia and the posterior aspect of the prostate (or alternatively, between the bladder and vagina in females). Bilateral isolation of the bladder is then accomplished, followed by the systematic transection of vesical pedicles after their secure clipping. The use of the fourth robotic arm facilitates optimal access to posterior plate and lateral pedicles, particularly in scenarios characterized by considerable tumoral burdens. After this step, an inverse U peritonectomy is meticulously executed between the two internal inguinal rings, following ligation of the umbilical arteries to facilitate access to the Retzius space. The anterior dissection of the bladder is carried out up to the Santorini complex. The urethra is carefully isolated and subsequently incised, following the placement of a Hem-o-lok clip to avoid urinary spillage. Frozen sections from the distal ureters and urethra are obtained. Afterwards, bilateral extended pelvic nodal dissection (LND) is performed; the dissection runs in proximity to the aortic bifurcation, presacral nodes, those adjacent to the common iliac vessels bordering the lateral margins of the genitofemoral nerves, nodes caudal to the circumflex iliac vein and the lacunar ligament, in addition to the Cloquet's node. A solitary case required a super-extended LND, consequent to the presence of macroscopically pathological nodes extending cranially to the level of the inferior mesenteric artery. The periureteral sheath is carefully preserved to maintain blood supply; ureters are mobilized only as much as required and each ureter is anastomosed on separate sides of the abdomen.

### Surgical technique for open cystectomy

Of the total cohort, 35 out of 42 patients (83%) underwent extraperitoneal radical cystectomy (RC). Pelvic lymph node dissection was performed in accordance with the previously described template. In certain cases, CUS was performed on the right side, with the left ureter transposed through the left colon mesentery to facilitate additional length.

### Variables and data collection

The included variables are depicted as follows:

- Pre-operative variables: baseline demographics (age, BMI), performance status assessed using the CCI and the ASA score.
- Post-operative variables: pathological stage (pT), transfusion rate, complications as graded by Clavien-Dindo, number of Clavien-Dindo ≥ 2 complications, and 30-day readmission rate.

Data were collected in a prospectively maintained Excel database; variables were entered from two Authors (E.P. and T.C.) who were not - or only marginally - involved in surgeries.

### Primary endpoint

The primary endpoint is to compare the rate of complication Clavien-Dindo ≥ 2 between ORC and RARC with CUS. The absolute number of complications per-patient is a secondary endpoint, as well as the complication rate (stratified as Clavien-Dindo ≥ 3).

## Statistical Analysis

Continuous variables were presented as median and interquartile range, whereas categorical variables were reported in terms of absolute and relative frequencies. To analyze differences in the distribution of continuous variables between open and robotic radical cystectomy groups, the Wilcoxon non-parametric rank-sum test was utilized. Fisher's exact test was employed to assess differences in proportions among the two study groups for categorical variables. To identify the factors associated with complications of Clavien-Dindo ≥ 2, a multivariable logistic model was estimated. For each factor, odds ratio (OR), 95% confidence interval (CI), and Wald test p-value were computed. Statistical analysis was also conducted after excluding pT4 cases, to eliminate instances potentially involving surgery-related complications due to complex or life-threatening dissections. All statistical analyses were performed using Stata version 16 (StataCorp. 2019. College Station, TX). A significant level of 5% was chosen for the analysis.

## RESULTS

Overall, 64 patients underwent RC with CUS, 42 (66%) with an open and 22 (34%) with a robotic approach. Among the latter, 2 cases underwent surgery with the Hugo RAS system. Overall, 49 (76%) males and 15 (24%) females were included. A descriptive analysis of the cohort stratified into robotic and open surgery is reported in Table-1. Baseline characteristics were similar between groups, except CCI score, which was significantly higher in the robotic group. Complications of Clavien-Dindo ≥ 2 occurred in 64.2% (27/42) of the open group and 13.6% (3/22) of the robotic group, respectively (p < 0.001); complications of Clavien-Dindo ≥ 3 occurred in 23.8% (10/42) and 4.5% (1/22) of the open and robotic group, respectively (p = 0.08). Open surgery was significantly associated with a higher number of complications. When excluding pT4 cases, complications ≥ Clavien-Dindo 2 occurred in 63.6% (21/33) and 10% (2/20) within open and robotic group, respectively (p < 0.001); complications Clavien-Dindo ≥ 3 occurred in 21.2% (7/33) and 5% (1/20) within open and robotic group, respectively (p = 0.2). Table-2 summarizes post-operative variables stratified by surgical procedure. Multivariable analysis revealed that robotic surgery was the only variable inversely associated with Clavien Dindo ≥ 2 complications (Table-3).

**Table 1:** Descriptive analysis of pre-operative variables stratified by surgical approach.

Variable		Open cystectomy (n=42)	Robotic cystectomy (n=22)	p
Age, years, median (IQR)		80 (67 – 85)	78 (69 – 85)	0.5
BMI Kg/m^2,^ median (IQR)		25 (22 – 27)	25.5 (24 – 28)	0.4
CCI, median (IQR)		7 (5 – 8)	8 (7 – 9)	0.04
**ASA score (n, %)**				0.3
	2	11 (26.8)	10 (45.4)	
	3	20 (48.8)	8 (36.4)	
	4	10 (24.4)	4 (18.2)	
**CCI (n, %)**				0.06
	3 – 6	20 (47.6)	3 (15.8)	
	7 – 8	13 (31)	10 (52.6)	
	> 9	9 (21.4)	6 (31.6)	

p = p-value from the Wilcoxon rank-sum test.

**Table 2:** Post-operative outcomes stratified by surgical approach. The data are shown as absolute frequencies with relative frequencies (in parentheses).

Variable (n, %)		Open cystectomy (n=42)	Robotic cystectomy (n=22)	p
**pT**				0.12
	pT1	12 (28.6)	9 (40.9)	
	pT2	4 (9.5)	6 (27.3)	
	pT3	17 (40.5)	5 (22.7)	
	pT4	9 (21.4)	2 (9.1)	
Transfusion rate		19 (45.2)	1 (4.6)	0.001
**Clavien complications (n, %)**				<0.001
	0 – 1	15 (35.7)	19 (86.4)	
	2 – 5	27 (64.3)	3 (13.6)	
				0.08
	0 – 2	32 (76.2)	21 (95.5)	
	3 – 5	10 (23.8)	1 (4.5)	
**No. of complications (n, %)**				0.005
	0	11 (26.2)	9 (42.8)	
	1	13 (30.9)	11 (52. 4)	
	≥2	18 (42.9)	1 (4.8)	
30-day re-admission		7 (17.5)	4 (18.2)	1

**Table 3:** Multivariable analysis of factors associated with Clavien-Dindo grade ≥ 2 complications.

		OR (95%CI)	p
Open cystectomy		**Reference**	
Robotic cystectomy		0.1 (0.02 – 0.5)	0.007
Age		0.99 (0.9 – 1.1)	0.80
**CCI 3 – 6**		**Reference**	
	CCI 7 – 8	0.54 (0.7 – 3.8)	0.54
	CCI ≥ 9	0.75 (0.08 – 6.9)	0.80
**ASA 2**		**Reference**	
	ASA 3	7 (1.0 – 47.3)	0.03
	ASA 4	10.6 (1.0 – 91.1)	0.99

OR = odds ratio; p= p-value from the Wald test for assessing the significance of the OR

## DISCUSSION

In the current series, the robotic approach to radical cystectomy with CUS provided advantages over open surgery in terms of transfusion and post-operative complication rate. The benefit particularly applies for complications Clavien-Dindo ≥ 2; furthermore, the number of such complications in the same patient is lower after RARC. Our outcomes are consistent with those of previous series and RCTs comparing robotic and open RC. While it is generally difficult and uncommon to conduct well-designed RCTs in surgery, research on radical cystectomy stands out. This field boasts a wealth of 8 RCTs, enabling precise comparisons between open and robotic RC. Khetrapal et al. (21) performed a systematic review and meta-analysis of perioperative, oncological, and quality of life outcomes from RCTs. By analyzing 8 trials accounting for 1024 participants, authors found that patients who underwent open cystectomy had higher rate of thromboembolic events (odds ratio [OR] 1.84, 95% CI 1.02–3.31, p = 0.04), incremented blood loss (MD 322 mL, 95% CI 193–450, p < 0.001) and transfusion rate (OR 2.35, 95% CI 1.65–3.36, p < 0.001). An extracorporeal realization of the diversion may mitigate a possible advantage on peri-operative morbidity that would have been expected for robotic RC. When addressing trials with only inclusion of intracorporeal diversions, the benefit in reduced venous thromboembolism and wound infection becomes even more evident; the lower use of ICU admission and a superior early recovery profile are advantages as well (22, 23). Provided that these benefits apply for complex reconstruction such as neobladder or ileal conduit, the use of robotic surgery for radical cystectomy is therefore expected to maintain its advantages also in candidates to a simple CUS. To our knowledge, this is the first study comparing robotic and open surgery in the setting of CUS as an elective indication. To now, robotic surgery for RC has been evaluated in frail patients with regards to age: two clinical studies addressed robotic surgery in octogenarian. Tanabe et al. (24) performed a retrospective cohort study on 74 patients, concluding that the incidence of perioperative complications of RARC in patients aged more than 80 years was not different from those in non-elderly individuals. Similar outcomes are reported by Chen et al. (25): authors compared 478 RARC cases in octogenarian with 2257 in a younger group. Complication rate, blood transfusion rate, and in-hospital mortality were similar to those in non-elderly. In the study, the authors also addressed the comparison between robotic surgery and other surgical approaches to RC and found that the RARC group had the lowest complication rate, and the shortest length of hospital stay. In our series, the use of robotic surgery in patients with a high CCI score (all patients were > 3) provided diminished blood loss, Clavien-Dindo ≥ 2 complications and a lower number of absolute complications Clavien-Dindo ≥ 2. We arbitrarily decided to address the number of complications as a separate variable, beyond their grade: unsurprisingly, 42.8% patients who underwent ORC experienced more than 2 complications, whereas a single patient (4.5%) of the robotic group had multiple adverse events. Furthermore, we addressed complication rate also after the exclusion of pT4 cases, those particularly prone to surgical complications due to the complexity of the surgical dissection. After excluding bulky bladder cancer, the rate of Clavien-Dindo ≥ 2 complications remains much lower in the robotic group compared to open surgery, confirming the less invasive fashion of robotics, which led to lower side effects.

Studying is not devoid of limitations. First, the single center fashion and the restricted sample size are main limits. Second, the absence of patient randomization. As aforementioned, the allocation of patients to the open or to the robotic arm was casual and driven by the surgeon's choice or local planning. Despite recognizing this limitation, it is worth noting that the ASA scores were similar between groups, and the CCI was even higher in the robotic cases. Third, there is a lack of consistent data related to length of stay (LOS). In our context of a public healthcare system, factors other than surgical complexity or complications, such as the time required to acquire self-stoma care skills and to organize outpatient services, often prolong LOS. Therefore, LOS data, influenced by these factors, could not be considered a reliable outcome measure. As a strength, it is noteworthy that this study is the first to explore the role of robotics in patients with an elective indication for CUS. Surgeons tend to select for robotic surgery only those patients who are suitable for complex surgery and/or have minor comorbidities. This series opens a new perspective, where the benefits of robotic surgery become increasingly apparent as patient frailty increases. The robotic cohort's low incidence of medical side effects (fewer thromboembolic events, less blood loss, lower wound infection rates) likely contributed to this finding. It is also important to note that the Trendelenburg position can be limited to 18° in RARC with CUS, thus broadening eligibility even for patients who cannot undergo a steep Trendelenburg position. From a technical perspective, RARC is marginally more complex than robotic assisted radical prostatectomy (RARP) and the operative times for RARC are like those for RARP. Thus, for surgeons experienced in RARP, the learning curve for the cystectomy component is minimal. This study has certain limitations, including a small sample size and the absence of long-term follow-up data.

## CONCLUSIONS

The current series confirms that robotic radical cystectomy is associated with lower morbidity, with reduced incidence of Clavien-Dindo ≥ 2 complications, and a lower number of adverse events. Therefore, robotic surgery appears to be particularly suitable for frail patients with an elective indication for cutaneous ureterostomy, where the benefits of a minimally invasive approach are more pronounced and could lead to a faster post-operative recovery.
